# Malignant Peripheral Nerve Sheath Tumor of Prostate: A Rare Case Report and Literature Review

**DOI:** 10.1155/2016/9317567

**Published:** 2016-10-30

**Authors:** Kun-Lin Hsieh, Chih-Cheng Lu, Chien-Feng Li, Yin-Hsun Feng, Alex C. Liao

**Affiliations:** ^1^Divisions of Urology, Department of Surgery, Chi Mei Medical Center, Liuying, Tainan, Taiwan; ^2^Department of Pathology, Chi Mei Medical Center, Tainan, Taiwan; ^3^Division of Hematology and Oncology, Department of Internal Medicine, Chi Mei Medical Center, Tainan, Taiwan; ^4^Divisions of Urology, Department of Surgery, Chi Mei Medical Center, Tainan, Taiwan

## Abstract

A mid-aged male presented with progressive lower urinary tract symptoms (LUTS) for years. Huge prostate with low serum prostate-specific antigen (PSA) level was detected. The specimen from transurethral resection revealed surprising pathology finding as malignant peripheral nerve sheath tumor (MPNST). Considering its huge size (more than 300 gm) and location, we prescribed neoadjuvant chemotherapy firstly. The tumor became regressive and then radical surgical resection was achieved. Adjuvant multimodality treatment including concurrent chemoradiotherapy (CCRT) and target therapy was given. However, he expired about one year later. MPNST originating from prostate is very rare and seldom reported before. We here present this extremely rare disease and share our treatment experience.

## 1. Introduction

Prostate cancer is one of the commonest malignancies in males, which is important to identify male patients who have enlarged prostate. Serum prostate-specific antigen (PSA) level is one of the key examinations to screen the malignancy potential. The majority of prostate cancer is adenocarcinoma, but there are still some uncommon malignancies developed from other origins [1]. Due to their nonacinar origin, the serum PSA level is typically not elevated. Here, we present a young male who came to urology outpatient department due to LUTS. Prostate survey revealed markedly enlarged prostate but very low PSA level. Biopsy pathology reported very-rare cancer: malignant peripheral nerve sheath tumor (MPNST) of the prostate.

## 2. Case Report

**Figure 1:**
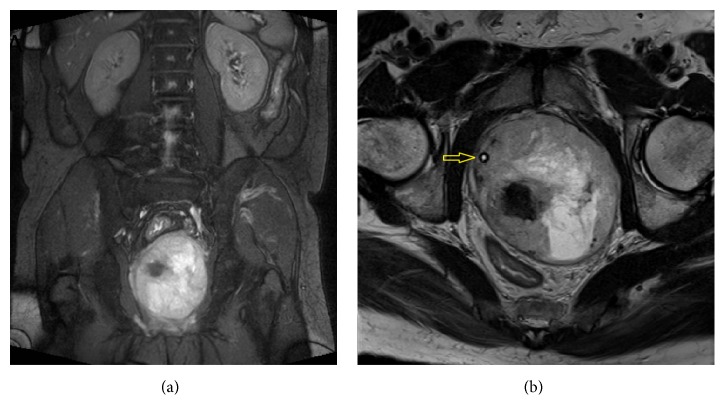
Prostate MRI with coronal (a) and axial (b) views showing marked enlargement of prostate gland with architecture derangement. Urethra with Foley (*arrow*) was shifted to the right side.

**Figure 2:**
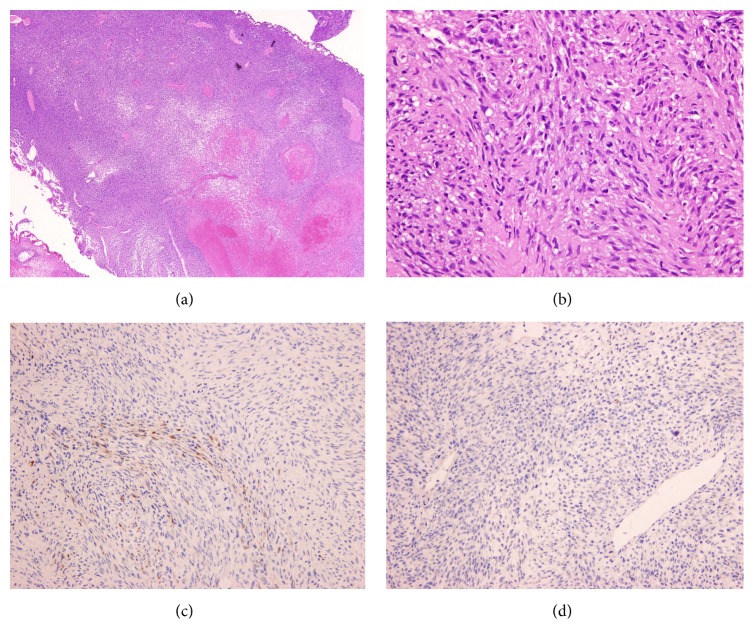
Microscopic findings of the transurethral resection biopsy specimen. (a) Low-power view shows spindle cells arranged in tightly packed fascicles, with alternating hypo- and hypercellular areas and geographic necrosis (H&E stain, ×40). (b) High-power view shows spindle-shaped tumor cells with hyperchromatic nuclei and frequent mitoses. Some entrapped prostatic glands are also seen (H&E stain, ×400). (c) By immunohistochemistry, the tumor cells demonstrate focal immunopositivity of S-100 protein (Immunoperoxidase, ×200) and (d) no immunoreactivity with CDK4 (Immunoperoxidase, ×200).

A 44-year-old man without systemic underlying disease suffered from painless gross hematuria combined with urine retention for days. He had LUTS for years and became worse recently. Stony-hard prostate with markedly enlarged size was found during digital rectal examination, but the PSA level was detected within the normal range (total PSA: 0.42 ng/mL). Transrectal ultrasonography (TRUS) revealed multiple nodules in the prostate. The largest nodule is 6.3 cm and the whole prostate measures about 300 grams. Architecture derangement of prostate, possible central necrosis with hemorrhage, and enlarged right internal iliac lymphadenopathy were found on prostate magnetic resonance imaging (MRI) (Figure 1). No bony metastasis was detected on the bone scan. We performed TRUS-guided prostate biopsy but massive gross hematuria attacked days after the procedure. Thus, cystoscopy for checking bleeding was done and the specimen resected from prostate was reported as malignant peripheral nerve sheath tumor (Figure 2). For further staging, we arranged chest computed tomography (CT), which revealed a solitary 0.9 cm nodule in the right upper lobe.

Considering the difficulty of radical surgery due to huge size of prostate, we prescribed neoadjuvant chemotherapy (5-Fluorouracil + Cisplatin + Ifosfamide) for 5 cycles. Regression of previous lung nodule was found on follow-up chest CT. Pelvic MRI revealed residual tumor lesion within the visible prostate gland, whose size regressed to 83 grams. The radical cystoprostatectomy was performed, and tumor invasion to bladder was found during operation. Bilateral pelvic lymph node dissection was also done and no metastasis was found. The pathologic diagnosis reported was consistent with MPNST.

After the operation, we prescribed adjuvant concomitant chemoradiation therapy (Cisplatin + 5-Fluorouracil + Ifosfamide with external beam radiation therapy 6600 cGy, 33 fractions). Follow-up abdominal CT done in postoperative 3 months showed multiple ill-defined poorly enhanced nodules in the liver and a 3.5 cm gallbladder mass. The target therapy, Pazopanib 400 mg per day, was given for palliative treatment due to suspect disease progression. However, his clinical condition went downhill and he expired three months later.

## 3. Discussion

MPNST is malignant tumor derived from peripheral nerves or demonstrated peripheral nerve differentiation. About half of the patients are associated with neurofibromatosis I (NF-I) and the others are sporadic or radiotherapy-induced. Some articles report prostate gland involvement in patients with NF-I, but most are benign lesions and originate from other pelvic organs [2]. MPNST developed from prostate is extremely rare. In our review, Rames and Smith presented the second case in 1999 [3], and no other case was reported except for our patient.

The most common clinical presentation is mass effect, which results from rapidly expanding tumor. Size is independent of the tumor site, but the average size is more than 5 cm. Besides, up to 50% of patients are diagnosed with metastasis status, where the most involved organ is lung. In our patient, progressive obstructive LUTS are the only symptom found. Besides, we noticed a small lung nodule at initial diagnosis, which is suspicious metastasis and regressed after neoadjuvant chemotherapy. Liver metastasis is also suspected in our patient. Thus, it is important to survey distant metastasis before and after treatment due to high metastasis rate.

Similar to other sarcomas, complete surgical resection is the treatment of choice. No randomized study strongly suggested chemotherapy to MPNST patient. However, in one systematic meta-analysis study, adjuvant chemotherapy for localized resectable soft-tissue sarcoma exists marginal survival benefit [4], implying that chemotherapy may have a role in selected MPNST patients. The benefit of neoadjuvant chemotherapy is also noticed in our presented case. Surgical approach became feasible due to regressive effect after neoadjuvant chemotherapy.

In recent years, many studies researched in molecular pathway about sarcomas. Several clinical trials about target therapy to sarcoma and MPNST are completed or ongoing, the results of which are exciting [5]. In PALETTE study, Pazopanib revealed a 3-month benefit in progression-free survival in patients with nonadipocytic sarcomas [6]. We prescribed the Pazopanib to this patient for palliative role due to progression, and he expired about 2 months after receiving Pazopanib. Further survey about target therapy on MPNST may be needed to study.

The MPNST is high-potential metastasis and the overall outcome is very poor. The 5-year overall survival rate is about 44% [7]. Larger size is related to higher local recurrent rate and distal metastasis potential [8]. Tumor site is another prognosis factor. Tumors located over extremities are more easily locally controlled, resulting in a better outcome. On the other hand, the neoadjuvant chemotherapy plays more important role in MPNST located in the pelvic cavity, where primary surgery may be hard to approach.

## 4. Conclusions

From our case and literature review, we learn that prostate MPNST is an extremely rare malignancy and still has no standard management protocol. Radical surgical resection is the key role of multimodality treatment; however, it may be infeasible due to large size and location. Our experience showed neoadjuvant chemotherapy is helpful for prostatic MPNST regression. However, even after radical surgery and adjuvant multimodality treatment, the prognosis is still poor. Target therapy is believed to become another promising treatment after further research in the future.

## References

[1] Paner G. P., Aron M., Hansel D. E., Amin M. B. (2012). Non-epithelial neoplasms of the prostate. *Histopathology*.

[2] Chung A. K., Michels V., Poland G. A., King B. F., Wojno K. J., Oesterling J. E. (1996). Neurofibromatosis with involvement of the prostate gland. *Urology*.

[3] Rames R. A., Smith M. T. (1999). Malignant peripheral nerve sheath tumor of the prostate: a rare manifestation of neurofibromatosis type 1. *The Journal of Urology*.

[4] Pervaiz N., Colterjohn N., Farrokhyar F., Tozer R., Figueredo A., Ghert M. (2008). A systematic meta-analysis of randomized controlled trials of adjuvant chemotherapy for localized resectable soft-tissue sarcoma. *Cancer*.

[5] Farid M., Demicco E. G., Garcia R. (2014). Malignant peripheral nerve sheath tumors. *The Oncologist*.

[6] van der Graaf W. T. A., Blay J.-Y., Chawla S. P. (2012). Pazopanib for metastatic soft-tissue sarcoma (PALETTE): a randomised, double-blind, placebo-controlled phase 3 trial. *The Lancet*.

[7] Kolberg M., Høland M., Agesen T. H. (2013). Survival meta-analyses for >1800 malignant peripheral nerve sheath tumor patients with and without neurofibromatosis type 1. *Neuro-Oncology*.

[8] Anghileri M., Miceli R., Fiore M. (2006). Malignant peripheral nerve sheath tumors: prognostic factors and survival in a series of patients treated at a single institution. *Cancer*.

